# Supplementary figures and images for: Recent phenological shifts of migratory birds at a Mediterranean spring stopover site: Species wintering in the Sahel advance passage more than tropical winterers
Source: PLoS One. 2020 Sep 18;15(9):e0239489. doi: 10.1371/journal.pone.0239489 (PMC7500615; doi:10.1371/journal.pone.0239489)

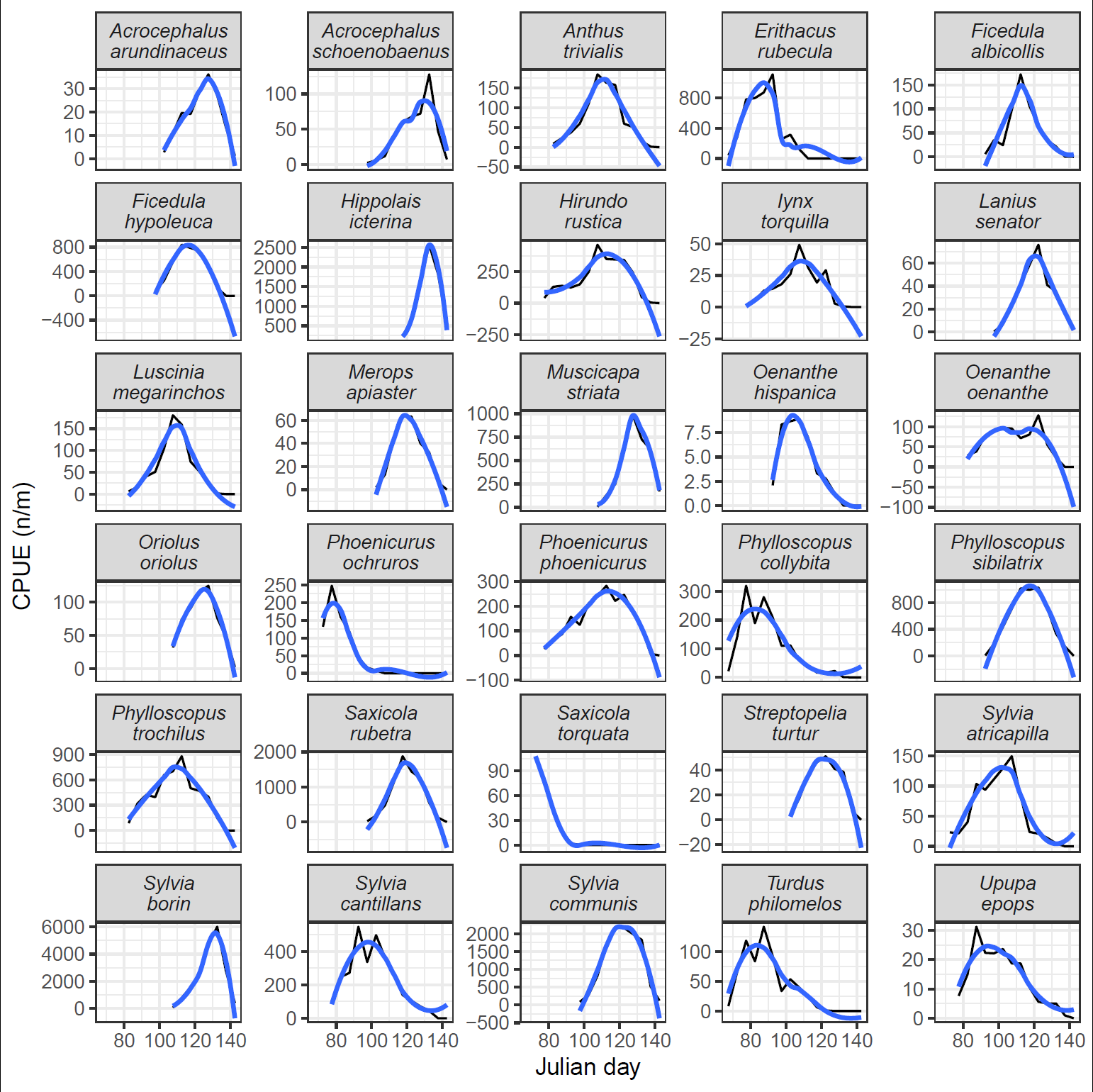

Supplement: S1 Fig — Average CPUE per day are represented by the black lines, while the moving average is represented by the blue curve. This figure only illustrates general patterns. Note, however, that peak, start, and end of the main migration period were calculated for every year separately for the analysis of timing patterns. (TIF) [file pone.0239489.s001.tif]

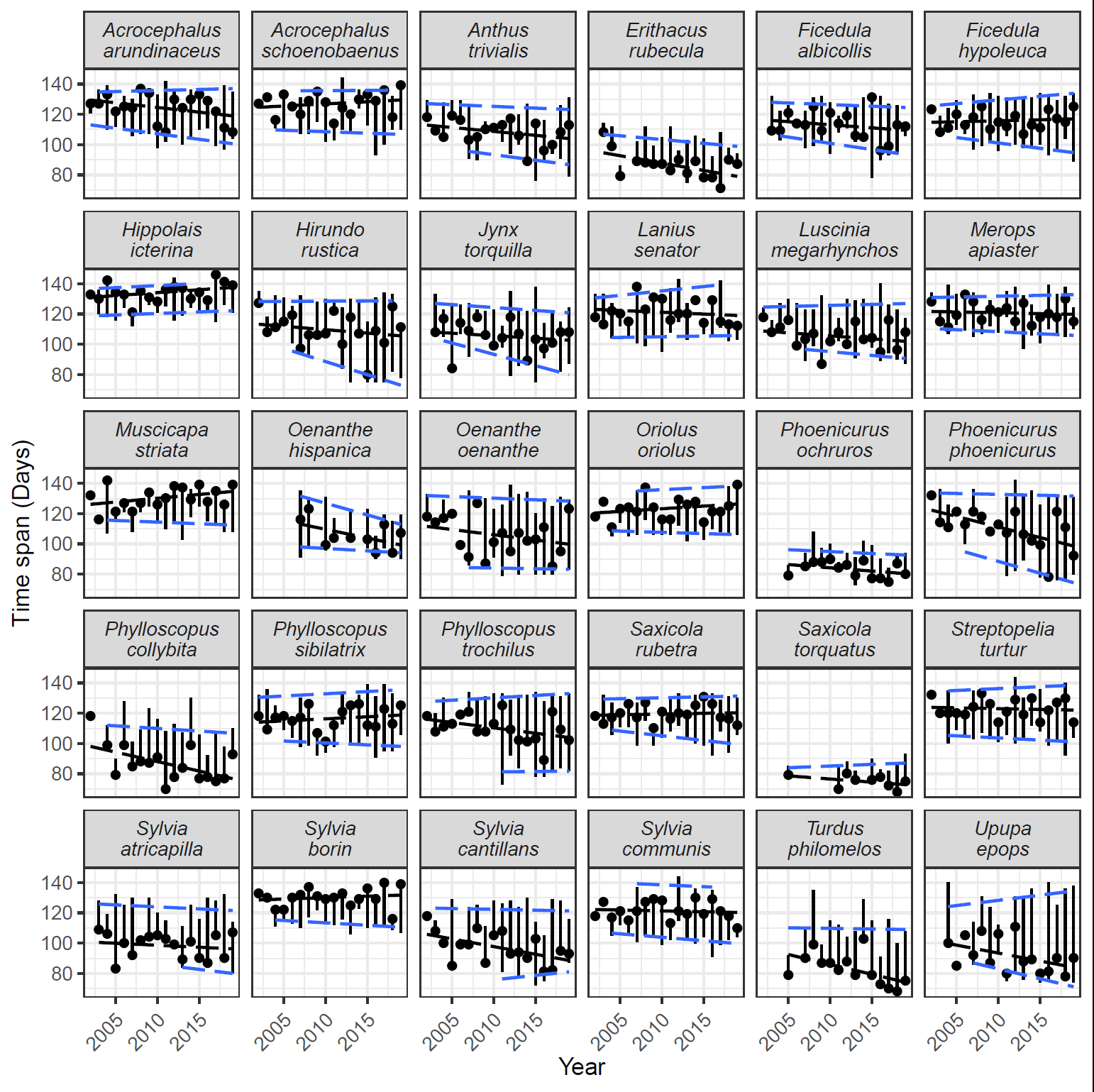

Supplement: S2 Fig — The blue lines represent the regression line of the start and end of the main migration period, while the black line represents the regression line for peak passage. Black dots represent yearly peak passage dates, while the whiskers represent start and end of the main migration period for every year of the study. (TIF) [file pone.0239489.s002.tif]
