# Supplementary material for: Recent phenological shifts of migratory birds at a Mediterranean spring stopover site: Species wintering in the Sahel advance passage more than tropical winterers
Source: PLoS One. 2020 Sep 18;15(9):e0239489. doi: 10.1371/journal.pone.0239489 (PMC7500615; doi:10.1371/journal.pone.0239489)
Supplement: S1 Table — (DOCX) [file pone.0239489.s003.docx]

**S1 Table. Start and end date of capture operations on Ponza during the 18 years of the study.**

| Year | Start | End |
| --- | --- | --- |
| 2002 | 28 April | 16 May |
| 2003 | 18 April | 17 May |
| 2004 | 8 April | 21 May |
| 2005 | 20 March | 14 May |
| 2006 | 9 April | 13 May |
| 2007 | 26 March | 18 May |
| 2008 | 28 March | 16 May |
| 2009 | 28 March | 15 May |
| 2010 | 27 March | 14 May |
| 2011 | 11 March | 23 May |
| 2012 | 18 March | 24 May |
| 2013 | 11 March | 17 May |
| 2014 | 30 March | 17 May |
| 2015 | 12 March | 22 May |
| 2016 | 13 March | 20 May |
| 2017 | 11 March | 26 May |
| 2018 | 9 March | 21 May |
| 2019 | 8 March | 19 May |
