# Supplementary material for: Recent phenological shifts of migratory birds at a Mediterranean spring stopover site: Species wintering in the Sahel advance passage more than tropical winterers
Source: PLoS One. 2020 Sep 18;15(9):e0239489. doi: 10.1371/journal.pone.0239489 (PMC7500615; doi:10.1371/journal.pone.0239489)
Supplement: S2 Table — Slopes and SE are shown in the main text in Table 2. (DOCX) [file pone.0239489.s004.docx]

**S2 Table. p-values of the linear regressions of passage date and year for 30 species migrating through Ponza between 2002 and 2019.** Slopes and SE are shown in the main text in Table 2.

| Species | Start | Peak | End | Migration window |
| --- | --- | --- | --- | --- |
|  |  |  |  |  |
| **Wintering group: North Africa** | | | | |
| *Erithacus rubecula* | NA | 0.023 | 0.241 | NA |
| *Phoenicurus ochruros* | NA | 0.204 | 0.571 | NA |
| *Phylloscopus collybita* | NA | 0.027 | 0.591 | NA |
| *Saxicola torquatus* | NA | 0.241 | 0.509 | NA |
| *Sylvia atricapilla* | NA | 0.526 | 0.479 | NA |
| *Turdus philomelos* | NA | 0.042 | 0.925 | NA |
| **Wintering group: Sahel** | | | | |
| *Acrocephalus schoenobaenus* | 0.644 | 0.401 | 0.978 | 0.733 |
| *Jynx torquilla* | 0.019 | 0.494 | 0.272 | 0.258 |
| *Lanius senator* | 0.765 | 0.510 | 0.231 | 0.158 |
| *Muscicapa striata* | 0.565 | 0.128 | NA | NA |
| *Oenanthe hispanica* | 0.428 | 0.123 | 0.014 | 0.102 |
| *Oenanthe oenanthe* | 0.863 | 0.232 | 0.450 | 0.888 |
| *Phoenicurus phoenicurus* | 0.016 | 0.008 | 0.655 | 0.826 |
| *Phylloscopus trochilus* | 0.959 | 0.077 | 0.093 | 0.971 |
| *Streptopelia turtur* | 0.286 | 0.725 | 0.610 | 0.549 |
| *Sylvia cantillans* | 0.614 | 0.024 | 0.812 | 0.053 |
| *Sylvia communis* | 0.120 | 0.682 | 0.767 | 0.553 |
| *Upupa epops* | 0.049 | 0.072 | 0.271 | 0.028 |
| **Wintering group: Tropical Africa** | | | | |
| *Acrocephalus arundinaceus* | 0.009 | 0.137 | 0.556 | 0.125 |
| *Anthus trivialis* | 0.291 | 0.154 | 0.465 | 0.581 |
| *Ficedula albicollis* | 0.135 | 0.375 | 0.453 | 0.504 |
| *Ficedula hypoleuca* | 0.023 | 0.618 | 0.013 | 0.074 |
| *Hippolais icterina* | 0.330 | 0.151 | 0.776 | 0.930 |
| *Hirundo rustica* | 0.006 | 0.391 | 0.924 | 0.020 |
| *Luscinia megarhynchos* | 0.276 | 0.302 | 0.627 | 0.421 |
| *Merops apiaster* | 0.270 | 0.718 | 0.719 | 0.374 |
| *Oriolus oriolus* | 0.444 | 0.307 | NA | NA |
| *Phylloscopus sibilatrix* | 0.525 | 0.417 | 0.216 | 0.159 |
| *Saxicola rubetra* | 0.083 | 0.714 | 0.375 | 0.315 |
| *Sylvia borin* | 0.235 | 0.515 | NA | NA |
