# Supplementary material for: Recent phenological shifts of migratory birds at a Mediterranean spring stopover site: Species wintering in the Sahel advance passage more than tropical winterers
Source: PLoS One. 2020 Sep 18;15(9):e0239489. doi: 10.1371/journal.pone.0239489 (PMC7500615; doi:10.1371/journal.pone.0239489)
Supplement: Attachment — Submitted filename: Response to reviewers_final_final.docx [file pone.0239489.s005.docx]

**Rebuttal letter to the manuscript**

PONE-D-20-09340
Recent phenology shifts of migratory birds at a Mediterranean spring stopover site: birds wintering in the Sahel advance passage more than tropical winterers
PLOS ONE

**NOTE**: responses are in ***bold italics*** characters.

**Editor comments**

Dear Dr. Maggini,

Thank you for submitting your manuscript to PLOS ONE. After careful consideration, we feel that it has merit but does not fully meet PLOS ONE’s publication criteria as it currently stands. Therefore, we invite you to submit a revised version of the manuscript that addresses the points raised during the review process.

The Reviewers made a great work and provided detailed and useful comments. Please consider all them because the suggested changes will add to the quality of the paper. In particular, I strongly advise you to consider with particular attention the comments regarding:

Reviewer #1

1. A formal comparison between different categories (long vs. short distance migrators, males vs. females)
2. Full reporting of all effects in statistical models, including the non-significant ones
3. Emphasizing the relevance of documenting an advancement in spring migration also in very recent years
4. Adding reference to recent literature (please note that the Reviewer very kindly provided a list of potentially relevant and recent papers)

Reviewer #2

1. Defend or discuss the statistical approach used to estimate phenology
2. Add details on the extent to which fieldwork could be performed according to the expected protocol

The major comment by Reviewer #2 deserves also consideration. Adding information on conditions at wintering sites or on passage areas across the Mediterranean would clearly add a lot of interest to the work.

***Dear Editor, we would like to thank the Reviewers for the careful review of our MS. We modified the paper according to their suggestions. Please see our point-by-point response to the reviewers below.***

Minor comments:

In Table 1 Oenante oenante is written with the species name capitalized. Please correct.

***Done.***

In line 126 and throughout the manuscript please replace “to anticipate” with “to advance”.

***Done.***

Journal Requirements:

When submitting your revision, we need you to address these additional requirements.

1. Please ensure that your manuscript meets PLOS ONE's style requirements, including those for file naming. The PLOS ONE style templates can be found at

<https://journals.plos.org/plosone/s/file?id=wjVg/PLOSOne_formatting_sample_main_body.pdf> and

<https://journals.plos.org/plosone/s/file?id=ba62/PLOSOne_formatting_sample_title_authors_affiliations.pdf>

***We adjusted the format of the manuscript to match the style requirements.***

2. In your Data Availability statement, you have not specified where the minimal data set underlying the results described in your manuscript can be found. PLOS defines a study's minimal data set as the underlying data used to reach the conclusions drawn in the manuscript and any additional data required to replicate the reported study findings in their entirety. All PLOS journals require that the minimal data set be made fully available. For more information about our data policy, please see <http://journals.plos.org/plosone/s/data-availability>.

Upon re-submitting your revised manuscript, please upload your study’s minimal underlying data set as either Supporting Information files or to a stable, public repository and include the relevant URLs, DOIs, or accession numbers within your revised cover letter. For a list of acceptable repositories, please see <http://journals.plos.org/plosone/s/data-availability#loc-recommended-repositories>. Any potentially identifying patient information must be fully anonymized.

***We made the dataset available at the URL:*** [***https://phaidra.vetmeduni.ac.at/view/o:114***](https://phaidra.vetmeduni.ac.at/view/o:114)***.***

Important: If there are ethical or legal restrictions to sharing your data publicly, please explain these restrictions in detail. Please see our guidelines for more information on what we consider unacceptable restrictions to publicly sharing data: <http://journals.plos.org/plosone/s/data-availability#loc-unacceptable-data-access-restrictions>. Note that it is not acceptable for the authors to be the sole named individuals responsible for ensuring data access.

***We have no restrictions to sharing the data.***

We will update your Data Availability statement to reflect the information you provide in your cover letter.

**Reviewers' comments:**

Reviewer #1: The study entitled “Recent phenology shifts of migratory birds at a Mediterranean spring stopover site: birds wintering in the Sahel advance passage more than tropical winterers” reports the description of the temporal variation in the dates of passage during the pre-nuptial migration of 18 bird species in a small island of the Mediterranean sea (Ponza). The main result is that most of the species under investigation advanced their passage time, and therefore add to the hundreds of studies reporting similar findings elsewhere in the globe. It also shows that species that migrate longer distances show a smaller temporal variation in phenology (e.g. the slope of the passage date against year) than those migrating further, as well as that males generally show steeper trends than females. The study is merely descriptive but worthy for a least two reasons: 1) it is conducted at an important passage site of which no information on migration time was available to date, and 2) because it was conducted in a rather recent period (2002-2019), and it therefore shows that birds are still shifting their phenology (and I think that this important result is not emphasized enough in the current version of the paper; please see below). I am therefore generally positive about the possibility that the paper would be accepted for publication. However, I am some issues that I would like to raise below in order to improve, I hope, the quality and the readability of the manuscript. I think that they should be easily addressed by the authors.

1) Statistical analyses and implications of the results. The conclusions are reasonable and compatible with the wide literature about the topic. However, I am not sure that the authors could claim that their study shows difference in the phenological variation between the species wintering in Northern Africa and in the Sahel zone and those wintering further South in the forests of central Africa without properly testing for it. I therefore encourage them adopt a proper statistical test in order to check for differences among groups of birds showing different migration strategies (and/or different wintering regions). This is also the case for the supposed differences between the phenology of males and females of some species. I am aware that the number of the species included in the analyses in not very large, but I think that there is the scope for splitting the species at least into two categories. However, if the authors think that this is not possible, in my opinion they should at least justify why they cannot analyse the data and add a caveat in the Discussion by acknowledging the reader about the limitations of their results.

***Our initial idea was to be conservative and not to categorize species, the main reason being the difficulty of assigning some of the species safely to a wintering area. We decided to leave room for interpretation and simply discuss the main trends without a formal analysis to avoid basing this analysis on wrong assumptions about the wintering area (e.g. some species could very well spend the winter in Northern and sub-Saharan Africa, e.g. Blackcap and Common Chiffchaff). However, we agree with the reviewer that the pattern we see strongly suggests an intriguing difference in phenological response between species wintering in different areas. Therefore, we followed the advice of the reviewer and assigned the species to three groups: species wintering mostly in North Africa, species wintering mostly in the Sahel zone (open scrubland, dry woodland), and species wintering mostly in tropical Africa (mostly forest). To assign species to each group we referred to Morel & Morel 1992 and Pearson & Lack 1992. Where there were discrepancies we assigned the species based on the range maps of Birds of the World (url:*** [***https://birdsoftheworld.org/bow/home?login***](https://birdsoftheworld.org/bow/home?login)***). Our results match very well the pattern that we recognized by investigating the species one by one. There are important and significant differences between the change in peak date of passage among the three groups. We also find differences between Sahel and Tropical wintering species: in the first, both males and females advanced their passage, while in the latter, only males did. We discuss these results in detail and rearranged the manuscript to better explain these results. We are confident that this additional analysis is justified, we think the results are very intriguing and we thank the reviewer for suggesting this.***

2) Main migration period. The study is focused on the start, end, duration and peak of the so-called ‘main migration period’. The procedure used to define this period seems accurate to me, and it is not my intention to question it. However, since the vast majority of the hundreds of studies on migration phenology published to date usually focus on mean/median and/or first (as well as last) passage dates, I am wondering why the authors opted for this different procedure, which make their results difficult to be compared with the previous ones. I would suggest to at least better justify this decision and to provide some solid reasons why this procedure is better, at least for the case under study, than that used in most of literature.

***This approach was chosen to deal with the problem of truncated sampling. In several species, we cannot be sure to have sampled the entire passage period, therefore defining quantiles makes little sense. We explain this with more detail in the text (lines 118-123). Several previous studies advised against using first and last arrival dates to determine the beginning and the end of the migratory period because they might overestimate changes due to strong outliers (e.g. Goodenough et al. 2015). Our Figure S1 shows visually that our estimates closely match the raw data, and therefore we believe that this is the most appropriate method to describe phenological changes.***

3) Tables. I have also a concern about how tables have been completed. Frankly, I do not agree with reporting only significant relationships in the tables. I think it is important to report all the associations, including non-significant ones. This good practice is important, for example, to limit publication bias in meta-analytic studies. I therefore strongly encourage the authors to fully fill the tables. In addition, since the main message of the paper is a link between migration strategy and phenological change, I also think that it would be very helpful for the readers (especially for those non-expert in birds) to have some additional information about the species in the first part of the manuscript (other than in the Discussion where they are now reported). A possibility is to add a column in Table 1 reporting the migratory behaviour and/or the main wintering region of each species.

***We agree with the reviewer and created more tables to add all relevant information. Table 1 now has general information about the species (including the wintering group they were assigned to), while Tables 2 and 3 have the slopes and SE for all linear regressions (including non-significant ones). Table 2 would have become too bulky if we also added all exact p-values, therefore we decided to underline significance levels using different typefaces and symbols, and reported all exact p-values in a new table in the Suppl. Mat. (Table S2).***

4) Introduction. On the whole, it is concise and well written. However, I was rather surprised not to read any clear mention about the effect of migratory behaviour on variation in migration phenology, also considering that the authors explicitly referred to short- and long-distance migrants in the Discussion. I understand that the authors mentioned where different species spend the wintering period, but information might not be enough for a generalist reader. According to literature, change in phenology depending on migratory behaviour is one of the most consolidated knowledge on the effect of climate change on birds. I would therefore encourage the authors to add a short paragraph on this (including relevant references), linked to that one focused on the effects of NAOI and wintering areas.

***We added a section about the different migratory behaviour to the introduction (lines 44-46, 60-63, 66, and 71-73), as suggested by the reviewer.***

5) Discussion. As briefly mentioned above, I think that one the main interesting point of the study is that migration data have been collected rather recently. I think that the paper would benefit from the inclusion of an additional paragraph where the authors emphasize that the phenological shifting is still ongoing and compare their recent trends with those obtained by previous studies performed on very long time-series (e.g. Askeyev et al. 2009. Climate Research, 38: 189-192; Kolářová et al. 2015. Climate Research, 63: 91-98).

***We now start the discussion mentioning that our results fill the recent lack of data on phenology, especially from this part of the migration route (lines 237-239). We agree that there are far less reports of changes in migration phenology in the recent literature.***

6) Literature cited. References quoted in the paper are generally relevant. However, I noted that only a few very recent papers (after 2015) have been cited, despite the vast literature produced in the last years. This is especially the case for Introduction, in which some very relevant and recent papers are missing. I therefore suggest to add some of them in order to better introduce the concepts, which will be developed through the manuscript. Please, find below some missing articles which are very relevant to the purpose of the study, and deserve to be cited (in chronological order).

- Kluen, E., Nousiainen, R., & Lehikoinen, A. (2017). Breeding phenological response to spring weather conditions in common Finnish birds: resident species respond stronger than migratory species. Journal of Avian Biology, 48(5), 611-619.
- Samplonius, J. M., Bartošová, L., Burgess, M. D., Bushuev, A. V., Eeva, T., Ivankina, E. V., ... & Mänd, R. (2018). Phenological sensitivity to climate change is higher in resident than in migrant bird populations among European cavity breeders. Global change biology, 24(8), 3780-3790.
- Ambrosini, R., Romano, A., & Saino, N. (2019). Changes in migration, carry-over effects, and migratory connectivity. Effects of Climate Change on Birds, 93.
- Radchuk, V., Reed, T., Teplitsky, C., Van De Pol, M., Charmantier, A., Hassall, C., ... & Avilés, J. M. (2019). Adaptive responses of animals to climate change are most likely insufficient. Nature communications, 10(1), 1-14.
- Horton, K. G., La Sorte, F. A., Sheldon, D., Lin, T. Y., Winner, K., Bernstein, G., ... & Farnsworth, A. (2020). Phenology of nocturnal avian migration has shifted at the continental scale. Nature Climate Change, 10(1), 63-68.

***We added novel references, including the ones cited by the reviewer, where relevant.***

Minor comments: LL 62-65. Maybe add some references here.

***We cite a few references on regional differences in the extent of the effects earlier (lines 44-45). Here, we present a prediction specific for our study species and study areas, and it would be difficult to make a link to previously published studies in a concise form.***

L 74. “near-passerine”. I think this should be defined, especially in a very generalist journal like the present one.

***We agree and we removed this term completely since irrelevant.***

LL 89-92 and Table 1. It is ok to include information about the total number of individuals captured per species. However, because the authors declared that they selected the species to be included in the analyses based on the yearly number of captures, I think that an important missing information is also the range of individuals captured per species per year. In addition, it is not clearly described which criterion was used to decide whether including or not a species. For example, what is the minimum number of specimens per year? In addition, did the authors exclude some years within species because they could not collect information on a minimum number of individuals? In my opinion, all this information should be added to improve the understanding of the procedures used.

***We added this information to Table 1. The criterion to include species was simply based on the abundance, and we included every species for which we had a sufficient sample every year (> 5 individuals/year).***

LL 113-115. Interesting that all the species show a significant temporal variation (advance or delay) in the phenology of migration. This is a quite unexpected result, if we compare it with the available literature. How do the authors explain this result? Is possible that the procedure used to identify the main migration period has somehow affected this result?

***We apologize, this was a mistake in the calculations in the previous version. We now corrected the calculations and there are far less significant slopes, though the slope sign is the same as in the original manuscript. However, following the advice of the reviewer we now moved the focus to the more general patterns by analyzing differences among groups of species with different wintering areas rather than a species-by-species analysis.***

L 244. Publication number XX. Maybe there is an error here.

***Publication number will be added upon acceptance of the manuscript.***

Reviewer #2: This paper aims to analyse the spring migratory phenology of 30 species of birds in a Mediterranean island, for a period of 18 years. Authors found a broadening of the time of passage overall, due to an advancement of the passage by mostly short-distance migrants.

The paper is well written, it is easy to follow and the concepts and literature used are within the expected current state of the art. A have, however, some comments to do:

Main comments:
The data set used in the study is impressive, and this is a robustness of the paper. However, the paper is totally descriptive, and the authors ‘only’ analyse a linear effect of year on the object, dependent variables (annual values of the start, peak and end of migration period). It would be really nice (though I acknowledge that this would entail much more work) if the authors may also look for the potential effects of conditions at wintering sites or during the passage across the Mediterranean. These analyses would allow a richer discussion around the observed changes/results. This is what I would also expect in a journal like PLOS ONE.

***We had several discussions about including environmental parameters to the analysis before submitting the manuscript. We decided against it for two main reasons: 1. The difficulty of identifying the exact wintering areas of the species analyzed; and 2. The consequent lack of novelty if we used global patterns. To 1. (our main point) we believe that the most interesting results of this manuscript (now even more strongly supported by the re-analysis suggested by reviewer #1) is the different extent of the change in timing between birds overwintering in different areas in Africa, and we agree that it would be very important to relate these results to environmental conditions in those areas. However, while assigning species to broad wintering areas is a relatively easy task, defining precisely what these areas are is more difficult and therefore environmental conditions would lack precision. We added a sentence regarding this issue to the discussion (lines 268-271). An analysis of global patterns would probably not add anything to the findings of dozens of previous studies that have focused on this aspect (our point 2). We believe that the main message of the paper is the existence of different responses of species wintering in different areas, which will stimulate further research on the causes of these differences, very likely to be environmental.***

Minor comments:
L94-96. Quite often, mist nets must be closed due to very adverse weather (heavy rain, too strong winds) or even logistical reasons (lack of personal in given moments). Even though the authors say that the fieldwork was done on a daily basis, I would like to know to what extent this protocol was done with a 100% of, most likely, whether there were some gaps. In these cases, many authors think that replacing raw data by theoretical distribution regressions is better, since these last allow to: deal with gaps in the data set and smooth the potential effects of the variables affecting capture rates (e.g., birds are more likely to be capture in days without wind, or in cloudy mornings as compared to very sunny days). Furthermore, these theoretical distributions would also allow dealing with years when the campaign started after the onset of migration, or ended before its end. Authors should discuss/defend their statistical approach and explain why not dealing with the use of theoretical distribution curves.

***See also response to reviewer #1 for the support of our statistical approach. We preferred not to use theoretical distribution curves because they are based on the assumption that the distribution of captures has a certain structure (mostly normal, or Poisson distributed). Our method allows an estimate that is closer to reality (see overlap in our smoothed data and the actual data in Fig S2). Despite losing information about the start of migration in North African wintering birds, we have peak passage dates for all species and this is the most important value, and can make nice inference about start and end of migration in Sahel and tropical overwinterers, which outline a relevant difference between these two groups. We agree that weather effects may cause oscillations in the number of captures that may be more related to the efficiency of capture than to the actual abundance of the species at the site. The extreme case would be when the effort of capture is forced to be zero because of the adverse conditions. However, using a smoothed curve like we did with our approach allows to level out these oscillations. Since the amount of days with no effort was less than 1% of the ringing days (and we have now added this information in the Methods section, lines 85-87), the overall effect of such days is negligible. In other days, the effort was reduced, but we account for that by using the CPUE (Captures per Unit Effort) instead of the absolute number of captures as a measure of daily abundance.***

L103. A good reason to use theoretical curves?

***In a previous analysis we also tried to estimate these data based on theoretical curves, with similar results to those presented here. However, we decided to refrain from that approach and to focus on the actual data. These are sufficient to recognize the important patterns and we believe that they are more robust than theoretical curves.***

L106-109. For me, this would be an insufficient explanation. There can be other distribution curves that might fit reasonably well to your data.

***We do not understand this comment. We state that our method is insensitive to the distribution of the data, therefore it does not require to estimate a distribution curve that fits to the data.***

L139. Please, this should be mention in Methods section.

***We moved this part to the Methods section, including a description of the new statistics applied as a follow-up to comments of reviewer #1 (lines 124-154).***

L157. Overall, the discussion might be benefited if the authors would also consider to test for the effects of conditions in the wintering/passing areas on their dependent variables.

***See our response to the main comment of the reviewer. The discussion has been largely rewritten to focus on the clear results obtained from the new analyses.***

Table 1. Please, add SE to the beta parameter estimtes (slope).

***These data are now all available in Tables 2 and 3.***

Table 2. Too many NAs in the start and end of migration. Why? Maybe this might be improved by using theoretical curves? If not, please limit the analyses to the peak passage parameter.

***We agree with the reviewer that the large number of NA’s (resulting from the approach that we used) renders this information relatively uninteresting. We therefore limited our analysis of the two sexes to the peak passage.***
