# Supplementary material for: Recent phenological shifts of migratory birds at a Mediterranean spring stopover site: Species wintering in the Sahel advance passage more than tropical winterers
Source: PLoS One. 2020 Sep 18;15(9):e0239489. doi: 10.1371/journal.pone.0239489 (PMC7500615; doi:10.1371/journal.pone.0239489)
Supplement: Attachment — Submitted filename: Response to reviewers revision.docx [file pone.0239489.s006.docx]

Reviewer #1: I am rather satisfied by the revision made by the authors because they provided all the main suggested changes to the manuscript. In my opinion, the paper has considerably improved and I therefore suggest it for publication.

***We thank the reviewer for the renewed assessment of our manuscript, and especially for her/his comments on the previous version that helped us to largely improve this new version.***

I have only a few additional minor comments (lines numbers refer to the version including track changes):

LL 151-152. Please add references used to define wintering areas and to associate species to them.

***The references are given in line 95 (new line numbering)***

L 211.5 rather than 22…a huge difference!

***We agree. This was due to a mistake in the previous analysis that we corrected in the new version. In the previous version, the large number of significant slopes caught the attention of the reviewers and was highly unlikely.***

LL 211-237. The reader is lost until the last line of this long paragraph because the table and the figure are cited only at end. I suggest to mention them before.

***We moved the reference to table and figure at the beginning of the paragraph.***

L 237. Fig. S2, not S2 Fig.

***This was suggested in the information for authors. We will change this if requested.***

Table 2. I am not sure that P values between 0.05 and 0.10 should be mentioned in a table, even considering that exact P-values are provided in the supplementary materials.

***We removed the highlights from slopes with p between 0.05 and 0.10 (we did the same in Table 3).***

LL 383-384. There is some evidence that in many species the advance in arrival date in the breeding area is less steeper than the passage date in the Mediterranean area. This point might deserve a short discussion and some references (please see below).
Both, C. (2010). Flexibility of timing of avian migration to climate change masked by environmental constraints en route. Current Biology, 20(3), 243-248.
Jonzén, N., Lind´en, A., Ergon, T., Knudsen, E., Vik, J.O., Rubolini, D., Piacentini, D., Brinch, C., Spina, F., Karlsson, L., Stervander, M., Andersson, A., Waldenstr¨om, J., Lehikoinen, A., Edvardsen, E., Solvang, R. & Stenseth, N. C. (2006). Rapid advance of spring arrival dates in long-distance migratory birds. Science 312, 1959–1961.
Bitterlin, L. R., & Van Buskirk, J. (2014). Ecological and life history correlates of changes in avian migration timing in response to climate change. Climate Research, 61(2), 109-121.

***We added a sentence to acknowledge this (l. 302-304).***

LL 390-391. A very important study to be cited here is: Dunn, P. O., & Møller, A. P. (2014). Changes in breeding phenology and population size of birds. Journal of Animal Ecology, 729-739.

***We added this reference.***
